# Gait Disturbances in Older Adults With Cerebral Small Vessel Disease: Mixed Methods Study Using Smartphone Sensors and Video Analysis

**DOI:** 10.2196/58864

**Published:** 2025-07-28

**Authors:** Xiaojun Lai, Li-Yan Qiao, Pei-Luen Patrick Rau, Yankuan Liu

**Affiliations:** 1 Department of Industrial Engineering, Tsinghua University, Beijing, China; 2 Department of Neurology, Yuquan Hospital of Tsinghua University, No. 5 Shijingshan Road, Shijingshan District, Beijing, 100040, China, 86 88257755

**Keywords:** cerebral small vessel disease, gait analysis, motor function assessment, cerebral, gait, motor function, mixed methods, mixed methods study, sensors, video analysis, video, tools, accelerometer, video data

## Abstract

**Background:**

Cerebral small vessel disease (CSVD) significantly impacts motor functions, particularly gait dynamics. However, its analysis often lacks the integration of comprehensive tools that capture the multifaceted nature of gait disturbances. Traditional methods may not fully address the complexity of CSVD’s impact on gait, underscoring the need for a detailed exploration of gait characteristics through advanced technological means.

**Objective:**

This study aims to identify the distinct gait patterns and postural adaptations present in patients with CSVD compared to a healthy older population, using an integrative analysis combining sensor and video data to provide a holistic understanding of gait dynamics in CSVD.

**Methods:**

This study involved 90 participants older than 50 years (mean age 68.85, SD 9.74 years; 47 males and 43 females), with 24 categorized as normal controls (mean age 66.42, SD 7.51 years) and 66 diagnosed with CSVD (mean age 69.74, SD 10.37 years). Participants performed three walking tasks: normal walking, dual-task walking (with concurrent mental arithmetic), and fast walking. Gait parameters were collected through video data for image posture parameters using the OpenPose BODY_25 key point model, and the “Pocket Gait Test” smartphone app for sensor-based parameters sampled at approximately 40 Hz. Data analysis included 5 sensor-based parameters (step frequency, root mean square (RMS), step variability, step regularity, and step symmetry) and 6 key video-based parameters (including knee angle, ankle angle, elbow angle, body trunk angles, and head posture).

**Results:**

Among the 29 participants with complete sensor and video data (10 normal controls and 19 patients with CSVD), significant differences were observed in step regularity (normal walking: mean 0.76, SD 0.09 vs mean 0.61, SD 0.25; *P*<.003 and dual-task: mean 0.74, SD 0.13 vs mean 0.57 SD 0.24*; P*<.005), RMS (normal walking: mean 1.64, SD 0.45 vs mean 1.43, SD 0.42; *P*<.006), and forward head posture angles (head-to-body angle during normal walking: mean 132.96, SD 7.78 vs mean 128.07, SD 7.99; *P*<.02 and head-to-ground angle: mean 134.11, SD 8.28 vs mean 128.40, SD 9.75; *P*<.008) between the CSVD and control groups. The CSVD group exhibited a more pronounced forward head posture across all walking tasks, with the greatest difference observed during dual-task walking (head-to-ground angle: mean 134.43, SD 8.29 vs mean 125.02, SD 8.42; *P*<.02).

**Conclusions:**

The study provides compelling evidence of distinct gait disturbances in patients with CSVD, characterized by reduced step regularity (15%‐23% lower than controls), altered acceleration patterns, and significant postural adaptations, particularly forward head positioning (4°‐7° more pronounced than controls). These quantifiable differences, detectable through accessible smartphone and video technology, offer potential biomarkers for early CSVD detection and monitoring. The integration of sensor and video analysis provides a more comprehensive assessment approach that could be implemented in both clinical and home settings for longitudinal monitoring of disease progression and rehabilitation outcomes.

## Introduction

### Background and Motivation

Gait analysis is commonly used to detect abnormalities in walking behavior, analyze physical function, and assess disease rehabilitation, indicating the occurrence of poor health problems or the progression of neurological diseases. Cerebral small vessel disease (CSVD) is characterized by brain white matter lesions, lacunar infarcts, and microbleeds on cranial imaging (especially head magnetic resonance imaging [MRI]) [1,2], and clinically can present as stroke, cognitive impairment, gait and balance dysfunction, emotional disorders, urinary disorders, etc [3,4]. CSVD has an estimated prevalence of 5%‐10% in the general population older than 50 years of age, with numbers increasing significantly with advancing age [5,6]. Recent evidence indicates that CSVD is the most common pathology underlying vascular cognitive impairment and is increasingly recognized as a primary contributor to gait disturbances in older adults [7,8]. Gait disturbance is a major and important clinical manifestation of patients with CSVD [9], but it is severely underestimated in clinical practice. There is an urgent need in clinical practice to intelligently collect, analyze, and report data on the gait of patients with CSVD and to diagnose and follow up on their condition.

Current assessment methods for CSVD-related gait disturbances are primarily limited to traditional clinical evaluations or laboratory-based systems that lack real-world applicability [3]. These existing approaches often fail to capture the subtle, multidimensional aspects of gait abnormalities specific to CSVD, particularly in everyday settings [10]. Recent systematic reviews and meta-analyses have highlighted that patients with CSVD commonly exhibit reduced walking speed, shorter stride length, increased stance time, and greater gait variability compared to healthy controls [3,8]. These gait characteristics are detectable even in early-stage CSVD and may precede cognitive symptoms, underscoring their potential value as early biomarkers. However, in the medical diagnostic process, many motor function impairments and gait disturbances associated with CSVD cannot be conveniently assessed using traditional cumbersome clinical tools. These motor manifestations, which often precede cognitive symptoms in patients with CSVD, require more accessible and efficient measurement approaches for timely diagnosis and monitoring.

Due to its convenience, remote application, and quantifiability, an intelligent wearable gait analysis measurement system offers a promising alternative direction in gait analysis. This approach directly addresses the limitations of conventional methods by enabling continuous, ecological monitoring of gait parameters in patients’ natural environments. Smartphone-based gait analysis has emerged as a valid and reliable method for capturing gait parameters, with recent validation studies demonstrating high agreement between smartphone accelerometry data and gold-standard systems in measuring key spatiotemporal gait parameters [10,11]. These smartphone-based systems can detect subtle gait abnormalities and have shown particular promise in neurological conditions where traditional assessment methods may be insensitive to early changes. On the one hand, medical costs are too high, and on the other hand, patients may delay diagnosis or treatment due to high medical expenses, requiring simpler and easier-to-use tools to assist in diagnosing diseases. In addition, the physical walking posture characteristics of patients at various stages, such as during illness and recovery, are significantly different, requiring tools that can effectively assist in assessing daily rehabilitation conditions over a long period. This study proposes combining image and sensor data to establish an intelligent wearable gait measurement system. This system allows for analyzing gait differences between patients with CSVD and healthy older groups. It also enables tracking of patient data during illness and recovery periods. Through this approach, key gait disturbances specifically related to CSVD can be identified. These findings can provide valuable reference points for the diagnosis of patients with CSVD and assist in their health monitoring and rehabilitation assessment.

This study aimed to conduct a detailed analysis of gait characteristics in patients with CSVD, using both sensor and video data. The objective was to identify and characterize distinct gait patterns and postural adaptations in patients with CSVD and to compare these with those of a healthy older population. Specifically, this study’s contribution includes the collection and analysis of real-world data from patients with CSVD. This effort sought to contribute to a deeper understanding of the impact of CSVD on motor functions, potentially guiding clinical assessments and rehabilitation strategies.

### Related Studies

Gait is the behavioral characteristic of human walking. Normal gait is the gait of a healthy adult walking in a natural state and feeling most comfortable, characterized by stable body posture, appropriate stride length, and minimal energy consumption [12]. Quantitative gait analysis is an important clinical indicator and, as a routine analysis in patient management, can be used together with medical history, physical examination, and other special investigations. Clinical gait analysis typically includes five elements: videotape examination, measurement of general spatiotemporal gait parameters, kinematic analysis, kinetic measurements, and electromyography [13]. Spatiotemporal gait data includes stride length, stride width, support time, rhythm, walking speed, swing time, and double-limb support time [14,15]. Gait parameters, including gait intensity, smoothness or regularity [16], variability, and complexity [17], can also be described by collecting acceleration signals during walking using specialized devices. Using kinematic (such as joint angles and angular velocities) and kinetic (such as ground reaction forces and joint torques) data, joint torques and forces can be calculated more precisely in 3D, and electromyography from specific muscles provides a detailed description of the gait mechanism [13,18].

Attributing gait abnormalities to a specific disease is difficult, as many diseases present similar gait abnormalities [19]. Special and precise quantification of gait abnormalities for specific diseases is needed. Diseases related to the cerebellum usually accompany specific gait differences, such as ataxic gait in spinocerebellar ataxia and cerebellar stroke. These conditions typically present with a wide-based stance, irregular stepping patterns, and poor coordination [20]. Similarly, CSVD has its own characteristic gait disturbances that require specific identification and quantification. Research on CSVD found that patients have reduced gait capabilities such as walking speed and stride length. De Laat et al [21] conducted MRI, voxel-based morphometry analysis, and walking ability assessments on 429 patients aged 50-80 years with CSVD but without dementia or Parkinson, finding a significant relationship between white matter lesions in CSVD and reduced gait ability, especially in terms of walking speed, stride length, and broad-based gait. In addition, they analyzed the relationship between the characteristic microbleeds of CSVD and gait ability using MRI to calculate the location and proportion of microbleeds, and using the GAITRite system, Tinetti gait scale, and Timed Up and Go measurements, finding that “microbleeds” resulted in shorter walking stride lengths and significantly lower Tinetti and Timed Up and Go scores [22]. Zong et al [23] used scales such as the ataxia rating scale, Tinetti gait score, Tinetti balance score, etc, for behavioral ability assessment and conducted gait and balance disorder studies on 57 patients with CSVD, finding that more than half of the participants showed gait disturbances such as reduced speed, dragging, wide base, and unequal bilateral stride lengths.

Traditional gait analysis methods are limited to fixed laboratory measurement environments, often using complex instruments such as single-camera image processing [24] and walking leap sensors [25] to record human gait motion, but they struggle to reflect real-world gait conditions. Moreover, nonwearable system equipment and testing are too expensive, making it difficult for people to measure related data on their own [26]. In recent years, portable, wireless real-time recording of daily gait information has become the main method of data collection. Zhong and Rau [27] used smartphones for gait assessment based on embedded accelerometers, finding that the reliability and effectiveness of smartphones in measuring gait parameters (stride length variability, autocorrelation, and root mean square [RMS] of acceleration) were comparable to external accelerometers. More recently, Tao et al [28] validated smartphone-based gait analysis across different attachment positions and walking speeds, including applications in patients with CSVD. Their study confirmed measurement validity in controlled environments, establishing that smartphones can provide reliable gait measurements comparable to traditional inertial measurement units. In addition, using OpenPose (developed by Cao et al [29] at Carnegie Mellon University) image recognition technology to collect key body metrics during walking and to calculate body posture parameters during walking can also serve as important gait feature data. At present, the key gait disturbances in patients with CSVD have not been fully identified, and there is ongoing research to establish a complete intelligent wearable gait measurement system based on smartphone-collected gait parameters, for use in assisting clinical diagnosis and rehabilitation monitoring. Other influencing factors, such as demographic information (age, sex, BMI, etc), disease severity, and other concurrent diseases, may also bring about changes or differences in gait. For example, gait abnormalities increase with age; men are more likely to have neurological gait abnormalities (such as stroke), while women are more likely to have non-neurological gait abnormalities (such as arthritis, cardiac, or respiratory diseases) [30]. Therefore, in addition to gait data, tracking and combining medical diagnostic results for in-depth research are needed to better assist in the diagnosis, assessment, and rehabilitation monitoring of diseases.

## Methods

### Overview

**Figure 1.**
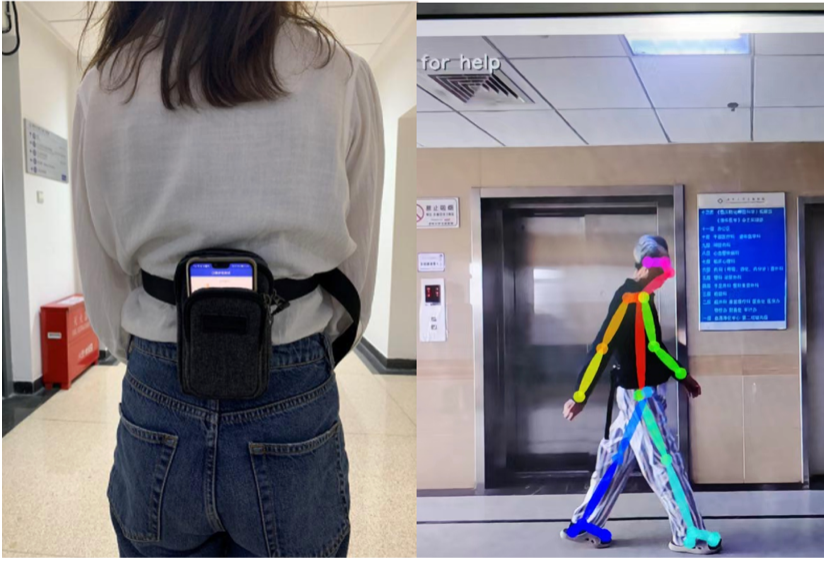
Field experimental environment setup and data collection method for gait assessment in patients with cerebral small vessel disease and healthy controls. (A) A participant wearing a waist bag containing a smartphone positioned at the L3 vertebra region for collecting accelerometer data and (B) the positioning of the GoPro HERO8 camera mounted on a tripod to capture a 5-meter walking segment.

This study was designed to assess gait characteristics in older adults with and without CSVD using a combination of sensor-based and video-based analysis methods. Participants completed three walking tasks—normal walking, dual-task walking, and fast walking—while wearing a smartphone equipped with accelerometers and being recorded by a camera. Data were collected on spatiotemporal gait parameters and postural angles to identify specific gait disturbances. The complete setup and data collection process are presented in Figures 1A and 1B.

### Recruitment

The study was conducted at Yuquan Hospital of Tsinghua University, a major medical center in Beijing, China, specializing in neurological disorders and geriatric care. Participants were not recruited through conventional research recruitment processes. Instead, a collaborative clinical screening approach was used. Physicians at Yuquan Hospital identified potentially eligible patients from their clinical practice who met the study criteria.

The recruitment process began with hospital neurologists reviewing medical records to identify individuals older than 50 years who either had a confirmed CSVD diagnosis or were free of neurological disease (for the control group). The physicians then conducted preliminary assessments of these individuals during their regular clinical visits to determine their suitability for the study based on mobility status and cognitive ability to follow instructions. After this clinical screening, suitable candidates were approached about potential participation in the study during their hospital visits. The physicians explained the study’s purpose, procedures, and potential benefits and risks. Those who expressed interest were provided with detailed written and verbal information about the study. All participation was strictly voluntary, and patients were assured that their decision regarding participation would not affect their clinical care.

For the CSVD group, inclusion was based on the presence of characteristic neuroimaging findings (white matter lesions, lacunar infarcts, or microbleeds on MRI) and clinical symptoms consistent with CSVD, as determined by the attending neurologists. Control group participants were recruited from the same hospital setting but were confirmed to be free of CSVD and other neurological conditions through clinical and radiological assessment.

This study involved 90 participants older than 50 years, of whom 24 participants were categorized as normal and 66 participants with CSVD. The groups were demographically comparable: the average age was 66.42 years in the normal group and 69.74 in the disease group (*P*=.14); sex distribution was 11 males to 13 females in the normal group and 36 men to 30 women in the disease group (*P*=.47). Heights and weights were also similar: normal group averaged 163.62 cm and 65.58 kg, while the disease group averaged 164.30 cm and 64.78 kg (*P*=.67 and .63, respectively). BMI showed no significant difference either, with 24.45 in the normal group and 23.81 in the disease group (*P*=.40). The demographic statistics for the participants are shown in Table 1.

**Table 1.** Demographic characteristics of participants in a cross-sectional observational study comparing gait parameters between patients with cerebral small vessel disease and healthy controls, conducted at Yuquan Hospital.

Variable	Normal, mean (SD)	CSVD^a^, mean (SD)	*P* value
Age (years)	66.42 (7.51)	69.74 (10.37)	.14
Height (cm)	163.62 (8.03)	164.30 (8.62)	.67
Weight (kg)	65.58 (9.47)	64.78 (12.91)	.63
BMI	24.45 (2.55)	23.81 (3.50)	.40

a CSVD: cerebral small vessel disease.

### Variables and Measurements

This study involved collecting gait parameters through accelerometer data for sensor-based parameters and video data for image posture parameters. Initially, 30-second walking data were gathered using the “Pocket Gait Test,” a gait measurement app developed by the authors’ research team (Zhong and Rau [27]). While other smartphone validation approaches, such as those by Tao et al [28], focus primarily on validating measurement accuracy, the “Pocket Gait Test” was specifically designed for analyzing subtle gait parameters in neurological conditions and older adults. This app uses the accelerometer embedded in smartphones and provides voice prompts to start and end the gait assessment, with a sampling rate of approximately 40 Hz. Custom algorithms developed in MATLAB (MathWorks) processed these data, resampling the discontinuous raw data to 100 Hz using interpolation.

Sensor gait parameters included step frequency, acceleration RMS, step variability, step regularity, and step symmetry. Step frequency refers to the rate of walking; RMS represents the magnitude of force generated during walking; step variability indicates the potential unsteadiness in gait, where lower values are preferable; step regularity refers to the consistency in walking pattern throughout; and step symmetry measures the difference between left and right leg movements, with higher symmetry indicating better gait balance.

Furthermore, walking videos covering approximately 5 meters were recorded using a GoPro HERO8 camera mounted on a tripod, set at a resolution of 1080p and a frame rate of 60 fps. The BODY_25 key point model was used with the OpenPose algorithm [29] to identify key body points and output JSON files for each video frame. This approach has been validated in previous human motion analysis studies [31-33]. Python was used to connect key points on the left (or right) side of the body into vectors and to process the videos in batches, following a methodology similar to that described by Viswakumar et al [31].

For signals with anomalies, especially when participants entered or exited the screen and key points were incomplete, parameters were adjusted to reselect video segments for analysis. MATLAB was then used for signal processing, using Db4 for three-level wavelet transform decomposition and reconstruction before data extraction. The output image posture parameters included the knee angle, defined as the angle between the thigh and calf segments; the ankle angle, representing the angle between the calf and foot segments; the elbow angle, indicating the angle at the elbow joint; the angle between the upper arm and body trunk, reflecting upper body posture; the angle between the body trunk and thigh, which reflects hip flexion or extension; the angle between the neck and body trunk, indicating forward head position; body lean, measured as the angle of the trunk relative to the vertical; sight line, representing the orientation angle of the head; heel height, defined as the vertical distance of the heel from the ground; step length, which is the distance between consecutive heel strikes; and step speed, which is derived from step length and time.

Particularly for KA, ankle angle, EA, the angle between the body trunk and thigh, and the angle between the upper arm and body trunk, which dynamically change with walking, the discussion focused on their mean and variance, while the analysis of the other parameters primarily concentrated on their mean values. The measurement of these angles follows standardized biomechanical conventions [34].

### Procedure

The experiment was conducted in the corridors of Yuquan Hospital. Before its commencement, participants’ age, sex, height, and weight were recorded. Their balance abilities and confidence were assessed using the Tinetti scale [35] and the Activities-Specific Balance Confidence (ABC) scale [36]. During the experiment, participants wore a waist bag containing a smartphone placed in the pocket at the L3 area of the waist to collect walking data for calculating sensor-based gait parameters.

Participants were instructed to perform three different walking tasks in order: normal walking, dual-task walking, and fast walking. These three tasks did not require specific speed requirements. For the first two tasks, participants were asked to walk at a natural and comfortable pace. The third task required participants to walk as quickly as possible. In addition, the second task involved a dual-task load, wherein participants were required to continuously perform mental arithmetic (addition and subtraction of two-digit numbers) while walking, to assess the impact of cognitive load on gait. Tasks were assigned randomly to eliminate the influence of the order of the experiment. Each condition was repeated twice. During the walking tasks, a GoPro HERO8 camera mounted on a tripod was positioned on the side of the corridor. This setup captured a 5-meter midsection of the participant’s walking path, including videos of both the left and right sides of the body.

The experimental setup controlled for variables independent of the video by managing the distance between the tripod and the participant, ensuring the participant occupied a consistent proportion of the video frame. This control allowed for the collection of consistent and reliable data across all participants and conditions. The field experimental environment is shown in Figure 1.

Illustration of the experimental setup at Yuquan Hospital corridors, where participants performed walking tasks while being recorded. The image shows (A) a participant wearing a waist bag containing a smartphone positioned at the L3 vertebra region for collecting accelerometer data, and (B) the positioning of the GoPro HERO8 camera mounted on a tripod to capture a 5-meter walking segment. Three walking conditions were tested: normal walking, dual-task walking (with mental arithmetic), and fast walking. Data were collected between January and September 2023 from 90 participants (24 healthy controls and 66 patients with CSVD), with complete data available for 29 participants (10 controls and 19 patients with CSVD).

### Data Exclusion

This study implemented a rigorous data exclusion process to maintain the quality and reliability of its results. After exclusions, the final analysis included 29 participants (10 normal controls and 19 patients with CSVD). The demographic characteristics of these participants are shown in Table 2. Similar to the initial recruitment cohort, these groups remained demographically comparable. This comparability ensures the validity of subsequent gait parameter comparisons between groups in the final analyzed cohort.

**Table 2.** Demographic characteristics of participants with complete data included in the final analysis of a cross-sectional study on gait parameters in cerebral small vessel disease.

Variables	Normal, mean (SD)	CSVD^a^, mean (SD)	*P* value
Age (years)	67.40 (6.79)	71.79 (7.56)	.12
Height (cm)	165.10 (9.65)	168.11 (7.65)	.29
Weight (kg)	66.10 (11.30)	65.13 (12.34)	.82
BMI	24.12 (2.23)	22.92 (3.22)	.37

a CSVD: cerebral small vessel disease.

Factors contributing to incomplete data included technical issues with sensor equipment (9.8% of exclusions), errors in video recording (37.7% of exclusions), and participants’ inability to complete all tasks due to physical limitations (29.5% of exclusions) or scheduling conflicts (26.2% of exclusions). In addition, data segments in which participants had just entered or exited the screen, resulting in incomplete key point detection by the OpenPose algorithm (approximately 10% of video recordings), were excluded to maintain consistency in the posture and gait analysis.

This stringent exclusion criterion was essential to ensure that the analysis was based on complete and accurate representations of each participant’s gait characteristics, thereby enhancing the study’s overall validity and reliability. While this reduced our effective sample size, it ensured that all analyzed data met the highest quality standards necessary for drawing meaningful conclusions about gait disturbances in CSVD.

### Ethical Considerations

This study follows the STROBE (Strengthening the Reporting of Observational Studies in Epidemiology) guidelines for cross-sectional studies and was conducted in accordance with ethical standards. Ethical approval was obtained from the Ethics Committee of Yuquan Hospital, Tsinghua University, and the Science and Technology Ethics Committee (Humanities, Social Sciences, and Engineering) of Tsinghua University. The formal IRB approval number is THU-04-2025-1023. All participants provided written informed consent before they participated in the study, which included consent for the collection, analysis, and publication of anonymized data and results. Participants did not receive financial compensation. The experiment was conducted on-site at Yuquan Hospital, where participants were already present for clinical care. Participation was entirely voluntary, with participants signing up to take part in the study of their own accord. All experimental procedures were strictly followed by the guidance of attending physicians to ensure participant safety and comfort. All assessments and data collection procedures were scheduled to accommodate participants’ daily routines and were conducted with careful attention to minimize fatigue, discomfort, or disruption to their normal activities. Their privacy and confidentiality were ensured throughout the study. All collected data were anonymized by assigning unique identification codes to each participant, and personal identifiers were stored separately from research data. During data analysis and reporting, no personally identifiable information was used, and results were presented as aggregated data. The video recordings and images used for gait analysis were processed using the OpenPose algorithm, which converts human figures into skeletal representations and removes identifiable facial features and body characteristics. No identifiable images of participants are included in the paper or supplementary materials. For the field experimental environment setup image (Figure 1), consent was obtained from the individual shown, and the image was processed to obscure any identifiable features.

## Results

### Sensor Data

Mann-Whitney *U* tests were used to compare gait characteristics between the normal and CSVD groups across various walking tasks, revealing several patterns. During a normal walking task, significant differences were noted in RMS (*P*=.006) and step regularity (*P*=.003), with the normal group having higher values, while step frequency, step variability, and step symmetry showed no significant differences between groups.

During dual walking conditions, RMS (*P*=.006) and step regularity (*P*=.005) again differed significantly, with the normal group showing higher values. In addition, step symmetry was significantly higher in the normal group (*P*=.001), while step frequency (*P*=.83) and step variability (*P*=.48) remained not significantly different.

In the fast walking condition, RMS (*P*<.001) and step regularity (*P*=.01) continued to show significant differences, with the normal group exhibiting higher values. Step frequency, step variability, and step symmetry did not differ significantly between groups.

These findings suggest that while certain aspects of gait, such as step frequency and variability, are not significantly affected by CSVD, other parameters, such as RMS and step regularity, consistently show noticeable differences across various walking conditions, with the CSVD group demonstrating significantly lower values than the normal group. Detailed sensor results are presented in Table 3.

**Table 3.** Comparison of sensor-based gait parameters between normal controls and patients with cerebral small vessel disease during three walking tasks.

Characteristic	Normal, mean (SD)	CSVD^a^, mean (SD)	*P* value
Normal walking			
Step frequency	1.75 (0.19)	2.00 (0.60)	.56
RMS^b^	1.64 (0.45)	1.43 (0.42)	.006
Step variability	0.10 (0.06)	0.12 (0.08)	.59
Step regularity	0.76 (0.09)	0.61 (0.25)	.003
Step symmetry	0.90 (0.06)	0.85 (0.15)	.11
Dual-task walking			
Step frequency	1.70 (0.17)	1.92 (0.43)	.82
RMS	1.57 (0.45)	1.28 (0.38)	.006
Step variability	0.10 (0.06)	0.13 (0.08)	.48
Step regularity	0.74 (0.13)	0.57 (0.24)	.005
Step symmetry	0.90 (0.09)	0.83 (0.15)	.001
Fast walking			
Step frequency	1.90 (0.18)	2.02 (0.42)	.58
RMS	2.17 (0.68)	1.86 (0.69)	<.001
Step variability	0.12 (0.06)	0.14 (0.06)	.72
Step regularity	0.80 (0.07)	0.63 (0.27)	.01
Step symmetry	0.94 (0.05)	0.81 (0.22)	.26

a CSVD: cerebral small vessel disease.

b RMS: root mean square.

### Video Data

Mann-Whitney *U* tests were used. Analysis was conducted on the mean angles of key body joints on both the left and right sides of 29 participants, focusing on the ankle, knee, elbow, thigh, head, and back. This analysis examined the changes in angles and angular accelerations of these joints across various tasks. The results indicated significant differences between the normal group and the CSVD group in three specific measurements: the mean angle between the calf and the vertical line from the ground, the angle between the head and the body, and the angle between the head and the ground.

Under normal walking conditions, significant differences were observed in the angles of the head with respect to the body (*P*=.023) and the ground (*P*=.008). The normal group had a mean head-to-body angle of 132.96 (SD 7.78) degrees and a head-to-ground angle of 134.11 (SD 8.28) degrees, compared to 128.07 (SD 7.99) degrees and 128.40 degrees (SD 9.75) degrees, respectively, for the CSVD group.

During dual walking, the CSVD group exhibited a significantly greater calf angle of 163.20 (SD 3.07) degrees compared to 159.97 (SD 2.40) degrees in the normal group (*P*=.004). The head-to-body (*P*=.021) and head-to-ground angles (*P*=.015) also showed significant differences, with the disease group demonstrating a more pronounced forward head posture.

In fast walking, although the calf angle did not differ significantly between the groups, the angles involving the head continued to show significant differences, with the CSVD group maintaining a more downward head position (head-to-body: *P*=.024; head-to-ground: *P*=.003).

These results suggest that patients with CSVD tend to lower their heads more when walking. This alteration in head posture could be indicative of balance or visual scanning changes in this population, potentially affecting their gait dynamics and overall mobility. Detailed video results are presented in Table 4.

**Table 4.** Comparison of video-based postural parameters between normal controls and patients with cerebral small vessel disease across three walking tasks.

Characteristic	Normal, mean (SD)	CSVD^a^, mean (SD)	*P* value
Normal walking			
Calf	159.81 (2.06)	161.54 (2.94)	.26
Head and body	132.96 (7.78)	128.07 (7.99)	.02
Head and ground	134.11 (8.28)	128.40 (9.75)	.008
Dual walking			
Calf	159.97 (2.40)	163.20 (3.07)	.004
Head and body	133.64 (8.83)	126.38 (7.08)	.02
Head and ground	134.43 (8.29)	125.02 (8.42)	.02
Fast walking			
Calf	159.26 (2.63)	160.91 (3.31)	.40
Head and body	133.66 (6.21)	128.39 (6.40)	.02
Head and ground	134.25 (6.20)	126.98 (7.75)	.003

a CSVD: cerebral small vessel disease.

## Discussion

### Principal Findings

This study has identified three key findings that significantly advance the understanding of gait disturbances in CSVD: (1) patients with CSVD exhibit a distinct forward head posture during walking tasks, with significantly different head-to-body and head-to-ground angles compared to healthy controls (2) step regularity and RMS values are significantly reduced in patients with CSVD across all walking conditions, suggesting altered gait dynamics and reduced stability; and (3) the integration of sensor and video data provides complementary insights that neither method alone could capture, demonstrating the value of a multimodal approach to gait analysis in CSVD. These findings collectively address the initial objective to identify and characterize distinct gait patterns and postural adaptations in patients with CSVD compared to healthy older adults, using the integrative analysis approach combining sensor and video data.

The forward head posture observed in patients with CSVD through video data might reflect adaptive mechanisms to maintain balance or compensate for proprioceptive deficits. This raises new questions about the nature of postural adaptations in CSVD. Notably, the sensor data provided precise measurements of these postural changes, while the video data offered a more qualitative understanding of the overall gait pattern. In contrast, the lack of significant differences in step frequency and variability between the normal and CSVD groups suggests that basic temporal aspects of gait might be less affected by CSVD. This finding is intriguing as it contrasts with some previous research [21], possibly due to methodological differences or the variability in disease severity among participants. It is important to consider that traditional gait metrics, while valuable, might not be sensitive enough to capture all aspects of gait disturbances in CSVD.

Furthermore, this study highlights the importance of considering both sensor and video data in gait analysis. The sensor data’s high precision in measuring joint angles and movements provides a detailed view of the mechanical aspects of gait, while video data offers a more holistic picture of gait, including body posture and coordination. This dual approach is crucial for a comprehensive analysis, as it captures both the quantitative and qualitative aspects of gait. The head angle, identified in this study as one of the differing factors in gait between patients with CSVD and healthy individuals, is challenging to capture through body sensors. The integration of video image processing enables the analysis of the overall gait in patients with CSVD, thereby providing more information that can be used to identify gait abnormalities in these individuals.

Integrating the clinical diagnosis of CSVD with the observed gait dynamics in patients with CSVD, this study underscores a critical linkage between early-stage CSVD manifestations and their impact on motor functions, as demonstrated by gait disturbances. The clinical findings from CSVD diagnosis reveal a pattern of gait disturbances that precede cognitive impairments, highlighting the significance of gait assessment in early CSVD identification. These disturbances, notably in dual-task walking conditions where patients exhibited lower average amplitude, gait regularity, and symmetry, align with observations of pronounced forward head posture in patients with CSVD through sensor and video analysis. This posture suggests an adaptation mechanism to maintain balance, potentially compensating for proprioceptive deficits intrinsic to CSVD. The convergence of clinical diagnosis with gait analysis in this study presents a compelling case for incorporating comprehensive gait assessment tools in the early detection and management of CSVD, fostering a holistic approach to patient care. The correlation between clinical indicators of CSVD and specific gait disturbances further supports the integration of sensor and video gait analysis into CSVD’s diagnostic and monitoring frameworks (refer to Table 5).

**Table 5.** Correlation between cerebral small vessel disease clinical findings and gait parameters in the observational study.

Parameter	Study finding	Clinical finding	*P* value (if applicable)
Forward head posture	Increase in patients with CSVD^a^	Confirmed	.008
Step regularity	Lower in patients with CSVD	Confirmed	.003
Step symmetry	Decrease in patients with CSVD	Confirmed	—
Head-to-body angle	Altered in patients with CSVD	Confirmed	.02
Gait disturbances	Identified	Confirmed	—

a CSVD: cerebral small vessel disease.

### Limitations

This study has several limitations that should be acknowledged. Although information was collected from a total of 90 participants, due to constraints such as experimental duration, scheduling, and task difficulty, complete data were obtained from only 29 participants (including sensor and video data across 3 tasks). This smaller number of participants could potentially impact the results to a certain extent and limit the ability to conduct more detailed subgroup analyses or to control for potential confounding factors such as age, sex, or comorbidities.

Technical challenges also affected the data collection. Approximately 9.8% of the exclusions were due to issues with sensor equipment, while 37.7% resulted from errors in video recording. In addition, 29.5% of participants were unable to complete all tasks due to physical limitations, and 26.2% had scheduling conflicts. These high exclusion rates highlight the practical challenges of conducting comprehensive gait analysis in older populations, particularly those with neurological conditions such as CSVD.

The OpenPose algorithm used for video analysis occasionally resulted in incomplete key point detection (approximately 10% of video recordings), particularly when participants were entering or leaving the screen. This necessitated careful selection of video segments for analysis, which may have introduced some selection bias. Furthermore, while our approach of combining sensor and video data provides complementary insights, it also introduces complexity in data integration and interpretation that may not be readily transferable to clinical settings without specialized expertise.

### Conclusions

This study offers valuable insights into the intricate gait disturbances experienced by patients with CSVD. By adopting a comprehensive approach that integrates sensor-based quantitative assessments with video-based qualitative observations, this study has enriched the understanding of CSVD’s impact on motor functions. The identification of specific gait parameters that differ between patients with CSVD and controls—particularly forward head posture and reduced step regularity—provides potential biomarkers for early detection and monitoring of CSVD progression.

These findings have several important implications for clinical practice and future research. First, they suggest that smartphone-based gait assessment combined with video analysis could serve as a cost-effective screening tool for early CSVD detection, potentially enabling earlier intervention before significant cognitive decline occurs. Second, the quantifiable nature of these gait parameters enables objective monitoring of disease progression over time, which could serve as outcome measures in clinical trials of new therapeutic interventions. Third, understanding the specific gait adaptations in CSVD suggests targeted rehabilitation approaches that might focus on improving proprioception and balance to address the forward head posture observed in these patients.

Furthermore, the smartphone-based assessment method demonstrated in this study has potential for remote monitoring applications, allowing clinicians to track patients’ gait parameters outside of clinical settings and providing a more ecological assessment of functional mobility. This approach could be particularly valuable in resource-limited settings or for patients with mobility restrictions that make frequent clinic visits challenging.

Future research should aim to validate these findings in larger, more diverse cohorts and explore the potential of longitudinal gait assessment to predict disease progression and response to interventions. In addition, the development of more user-friendly, automated analysis tools could facilitate the integration of comprehensive gait assessment into routine clinical care for patients with CSVD, potentially improving early detection, monitoring, and personalized treatment planning for this common but often underdiagnosed condition.

## Abbreviations

ABC: Activities-Specific Balance Confidence
CSVD: cerebral small vessel disease
MRI: magnetic resonance imaging
RMS: root mean square
STROBE: Strengthening the Reporting of Observational Studies in Epidemiology
